# Unravelling the Molecular Epidemiology and Genetic Diversity among *Burkholderia pseudomallei* Isolates from South India Using Multi-Locus Sequence Typing

**DOI:** 10.1371/journal.pone.0168331

**Published:** 2016-12-19

**Authors:** Chaitanya Tellapragada, Aayushi Kamthan, Tushar Shaw, Vandana KE, Subodh Kumar, Vinod Bhat, Chiranjay Mukhopadhyay

**Affiliations:** 1 Department of Microbiology, Kasturba Medical College, Manipal University, Manipal, India; 2 Division of Microbiology, Defense Research and Development Organization, Gwalior, India; 3 Department of Community Medicine, Kasturba Medical College, Manipal University, Manipal, India; Institut National de la Recherche Agronomique, FRANCE

## Abstract

There is a slow but steady rise in the case detection rates of melioidosis from various parts of the Indian sub-continent in the past two decades. However, the epidemiology of the disease in India and the surrounding South Asian countries remains far from well elucidated. Multi-locus sequence typing (MLST) is a useful epidemiological tool to study the genetic relatedness of bacterial isolates both with-in and across the countries. With this background, we studied the molecular epidemiology of 32 *Burkholderia pseudomallei* isolates (31 clinical and 1 soil isolate) obtained during 2006–2015 from various parts of south India using multi-locus sequencing typing and analysis. Of the 32 isolates included in the analysis, 30 (93.7%) had novel allelic profiles that were not reported previously. Sequence type (ST) 1368 (n = 15, 46.8%) with allelic profile (1, 4, 6, 4, 1, 1, 3) was the most common genotype observed. We did not observe a genotypic association of STs with geographical location, type of infection and year of isolation in the present study. Measure of genetic differentiation (F_ST_) between Indian and the rest of world isolates was 0.14413. Occurrence of the same ST across three adjacent states of south India suggest the dispersion of *B*.*pseudomallei* across the south western coastal part of India with limited geographical clustering. However, majority of the STs reported from the present study remained as “outliers” on the eBURST “Population snapshot”, suggesting the genetic diversity of Indian isolates from the Australasian and Southeast Asian isolates.

## Introduction

Melioidosis, caused by soil saprophytic bacteria *Burkholderia pseudomallei* is a fatal infection among human and animals of the tropics. Numerous factors such as the amount of rainfall, soil types based on texture, pH, salinity and moisture content were reported to influence the presence of *B*.*pseudomallei* in soil[1]. Exposure to this pathogen via inoculation, inhalation or ingestion can lead to diverse clinical manifestations and high case fatality, if left untreated. Since the time of its first description over a century ago, the disease was thought to be restricted between 20^0^N and 20^0^S of the equator, with the highest incidence in Southeast Asian countries and Northern Australia[2]. However, there is mounting evidence suggesting the occurrence of melioidosis in numerous countries such as the Sub-Saharan countries of Africa, Middle East, Brazil and China over the past few decades[3]. In India, melioidosis is slowly gaining recognition as an emerging bacterial infection after decades of being severely underreported or misdiagnosed. Lack of distinct clinical pathognomic features among the infected and limited utility of conventional microbiological culture techniques for detection of this pathogen can be attributed for this underreporting. When attempts were made to map the cases of human melioidosis published in the literature from India, it was evident that the disease is predominately reported from tertiary care microbiology laboratory settings located in the coastal parts of the sub-continent. Moreover, there was no consistency observed in the form of melioidosis (clinical presentations) reported among these sporadic cases across the country [4–6]. Given this context, questions such as the following arise: a) whether the disease is restricted to a few geographical hot spots in the country or is it ubiquitously present? b) Is melioidosis endemic in India and if endemic, can it be isolated from the soil? c) Are the Indian isolates genetically diverse from the Southeast Asian and Australasian isolates? d) Is there a geographical variation in the clinical presentations of the disease due to circulating genotypes of *B*.*pseudomallei*?.

With this background, we aimed at studying the genetic diversity among Indian *B*.*pseudomallei* isolates of both clinical and environmental origin from those isolated from the rest of world using Multilocus sequence typing (MLST). As an additional outcome we also looked for an association between the circulating *B*.*pseudomallei* sequence type (ST) and the form of the disease among the infected.

## Materials and Methods

### Study isolates

A total of 32 non-repetitive *Burkholderia pseudomallei* strains with clinical (n = 31) and environmental (n = 1) origin isolated between 2006 and 2015, from various parts of Karnataka and its adjacent states of southern India were included in the study. The study was approved by Institutional Ethical committee, Kasturba Hospital Manipal. All the isolates were confirmed as *B*.*pseudomallei* using standard biochemical tests and later confirmed using TTS1 PCR and latex agglutination test from the colonies.

### DNA extraction

Genomic DNA was isolated in a category III biocontainment facility at the Defence R & D Establishment, Gwalior. Overnight bacterial cultures were inoculated from freezer stock and grown in Luria-Bertani (LB) broth at 37°C. Genomic DNA was isolated using the Dneasy blood and tissue genomic DNA kit (Qiagen), according to the manufacturer’s instructions. Genomic DNAs were stored at -20°C for further use.

### Multilocus sequence typing

#### a. Amplification of the housekeeping genes

For the performance of MLST, primers targeting the highly conserved regions of seven housekeeping genes of *B*. *pseudomallei* were employed. Established oligonucleotide primer sequences reported previously for MLST of *B*.*pseudomallei* were used[2]. The amplification mixture (total volume of 30μl) contained 3μl of 10X PCR buffer (Qiagen) with15mM MgCl_2_; 3μl of 2mM dNTP (Qiagen); 2μl of 5X Betaine solution, 0.2μl of 5U TaqDNA polymerase (Sigma); 3μl of 10pmol primers and 50 ng of purified bacterial DNA. Amplification was carried out in a Master cycler gradient (Eppendorf, Hamburg, Germany) with an initial denaturation at 95°C for 4 min, followed by 30 cycles of 95°C for 30s, 62°C for 30s, and 72°C for 60s followed by a final extension step of 72°C for 10 min. Amplicons were visualized in 2% agarose gel incorporated with 0.5% ethidium bromide under a UV transilluminator.

#### b. DNA purification, sequencing and analysis

Amplicons were purified with QIAquick PCR purification kit. The purified PCR products were analyzed via 1% agarose gel electrophoresis for sufficient product, correct size, and product purity. Purified amplicon DNA were submitted for SANGER sequencing to the Genotypic Technology Pvt Ltd. Bangalore. Each DNA fragment was sequenced in both forward and reverse directions. For the sequence analysis of each DNA fragment, the forward and reverse sequences were aligned with a reference allele sequence obtained from the *B*. *pseudomallei* MLST website (www.http://bpseudomallei.mlst.net/) using the ClustalW2-Multiple Sequence Alignment-EMBL-EBI (www.ebi.ac.uk/Tools/msa/clustalw2). DNA sequences were edited using DNA Baser Sequence Assembler v4 (Heracle BioSoft, www.DnaBaser.com). Sequences obtained for each of the seven loci of individual bacterial isolates were given an allelic number using the online software (www.http://bpseudomallei.mlst.net/). A string of seven integers (allelic profile) denoting the allelic number of each locus *(ace- gltB- gmhD- lepA- lip- narK- ndh*) was obtained. The allelic profile of each isolate was queried for a match to the existing sequence types (ST) on the MLST database. Multilocus sequence analysis (MLSA) was performed using eBURST with single-locus variant (SLV) selected. Measure of genetic differentiation (F_ST_) between the concatenated sequences of Indian origin and the STs from the rest of world was estimated using DNAsp V5.1 as described previously[7]. Further, Fisher’s exact t- test was used to find the association of individual ST with type of infection among the study population.

## Results

**Fig 1:**
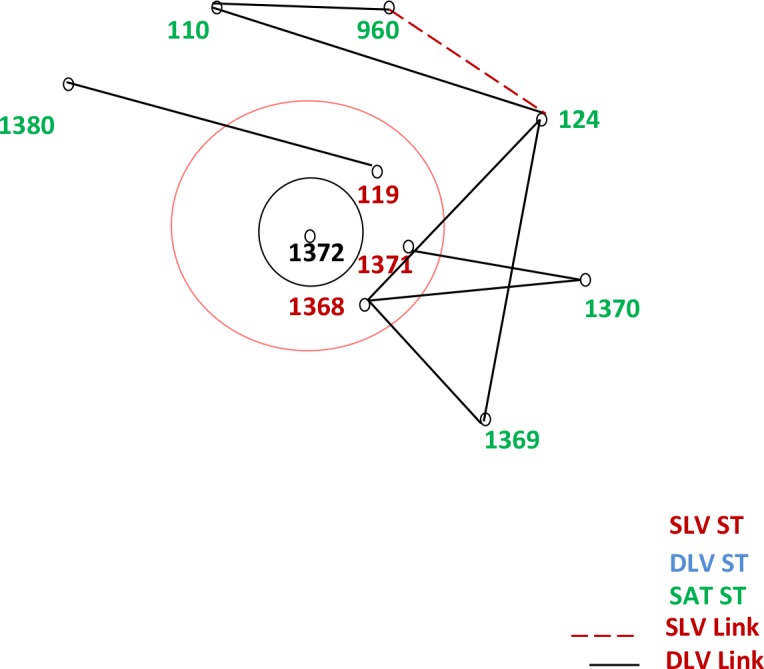
Genetic relatedness of the present study isolates using eBURST.

**Fig 2:**
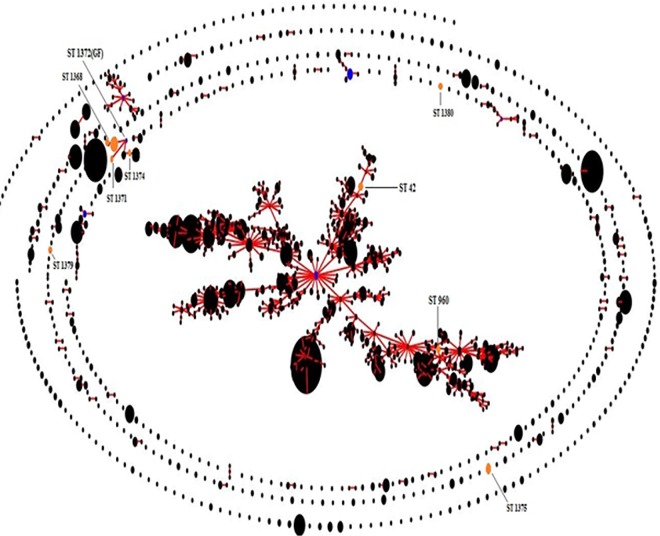
Population snapshot of all B.pseudomallei isolates in the MLST database including the present study isolates.

Among the 31 clinical isolates, 20(64.5%) were obtained from patients with localized form of melioidosis and 11 (35.5%) from patients with bacteremic melioidosis. Majority (n = 26, 81.2%) of the study isolates were isolated from the state of Karnataka, India. Distribution of the study isolates based on the clinical condition, geographical location, year of isolation are enlisted below (Table 1).Of the 32 isolates included in the analysis, 30 (93.7%) had novel allelic profiles that were not reported previously. Overall, 14 STs were observed in the present study with 12 of them being novel. ST 1368 (n = 15, 46.8%) with allelic profile (1, 4, 6, 4, 1, 1, 3) was the most common genotype observed. We did not observe a genotypic association of STs with geographical location, type of infection and year of isolation in the present study. Details regarding the year of isolation, specimen and type of infection for all STs is enlisted below (Table 1). Using eBURST, the 32 study isolates were grouped in to 1 lineage with 4 singleton sequence types (Fig 1 and Table 2) and ST 1372 as the founder ST. Nucleotide diversity (π) among the Indian isolates was 0.00190. STs from the present study were all outliers to the STs from the rest of world except for ST 1372 (group founder of the present study isolates), which was a double- locus variant (DLV) of Sri Lankan STs 1134, 1138 and 1140. “Population snapshot” of all *B*.*pseudomallei* isolates in the MLST database including our study isolates is depicted below (Fig 2). Measure of genetic differentiation (F_ST_) between Indian and the rest of world isolates was 0.14413. We did not observe a significant association of individual STs with the form of the disease presentation (*P* = 0.593) among the study population.

**Table 1:** Description of the study isolates based on type of infection, geographical location of isolation, year of isolation and the sequence types.

Isolate No	Type of infection	Specimen	Geographical location Place, State	Year of Isolation	Sequence Type
Ma23	Localized	Pus	Ankola, Karnataka	2010	1372
Ma10	Systemic	Bone marrow	Manipal, Karnataka	2009	1370
Ma16	Localized	Pus	Bhatkal, Karnataka	2009	1368
Ma86	Localized	Pus	Chikmanglur, Karnataka	2010	1368
Ma85	Systemic	Blood	Chitradurga, Karnataka	2008	1368
Ma19	Systemic	Blood	Chitradurga, Karnataka	2009	1375
Ma24	Pulmonary	Sputum	Davangere, Karnataka	2010	1368
Ma87	Systemic	Blood	Goa, Goa	2014	1368
B19522	Localized	Pus	Hyderabad, Telangana	2015	1379
E14922	Localized	Pus	Hyderabad,Telengana	2015	1379
Ma8	Localized	Pus	Karwar, Karnataka	2008	1371
Ma20	Systemic	Blood	Kaup, Karnataka	2010	1368
Ma143	Systemic	Blood	Cochin, Kerala	2015	1380
Ma1	Systemic	Blood	Cochin, Kerala	2006	1368
Ma4	Systemic	Blood	Cochin, Kerala	2007	1368
Ma18	Systemic	Blood	Kumta, Karnataka	2009	1368
Ma2	Localized	Pus	Mangalore, Karnataka	2007	1369
Ma5	Localized	Pus	Manipal, Karnataka	2008	1370
Ma3	Localized	Pus	Shimoga, Karnataka	2007	1368
Ma17	Localized	Pus	Shimoga, Karnataka	2009	1368
Ma21	Systemic	Blood	Shimoga, Karnataka	2010	1375
Ma81	Localized	Pus	Shimoga, Karnataka	2014	124
Ma82	Localized	Pus	Shimoga, Karnataka	2015	133
Ma88	Localized	Pus	Udupi, Karnataka	2010	1368
Ma89	Pulmonary	BAL	Udupi, Karnataka	2010	1368
Ma9	Localized	Pus	Udupi, Karnataka	2008	1368
Ma15	Localized	Pus	Udupi, Karnataka	2009	1371
Ma29	Localized	Synovial fluid	Uttarkannada, Karnataka	2010	1368
Ma83	Systemic	Blood	Uttarkannada, Karnataka	2014	42
Ma84	Localized	Pus	Uttarkannada, Karnataka	2015	960
Ma80	Localized	Pus	Uttarkannada, Karnataka.	2014	110
MaS1	Soil	Soil	Kemmanu, Karnataka	2015	119

**Table 2:** Description of the study isolates based on eBURST analysis.

ST	Frequency	Single locus variants	Double locus variants	Satellites
1368	15	1	5	3
960	1	1	1	7
1380	1	0	1	8
1369	1	0	2	7
1370	2	0	2	7
1371	2	1	3	5
1372*	1	3	0	6
119	1	1	3	5
110	1	0	2	7
124	1	1	3	5
42**	1	-	-	-
1379**	2	-	-	-
1375**	2	-	-	-
133**	1	-	-	-

*Founder ST

** Singleton STs.

## Discussion

Burkholderia pseudomallei, as an etiological cause of community acquired septicemia and pneumonia has gained recognition in India over the past two decades. Numerous molecular typing methods such as PCR-RFLP, ribotyping and pulse-field gel electrophoresis were reported previously with limited utility in studying the epidemiology of melioidosis. Multilocus sequence typing is well accepted as a useful epidemiological tool to study the biogeographic and phylogenetic aspects of bacterial pathogens such as *B*.*pseudomallei* due to its robustness, reproducibility and ease to perform [2]. Further, analysis of MLST data using a phylogenetic tool such as the eBURST has succeeded in elucidating the genetic relatedness of *B*.*pseudomallei* from distinct geographical locations and more importantly the population- level trends [8, 9].In our quest to study the molecular epidemiology of *B*.*pseudomallei* isolates from India, we previously reported the MLST and MLVA-4 results of seven clinical isolates of *B*.*pseudomallei* from patients diagnosed with melioidosis at our settings[10]. In the present study, we furthered our research by including 32 *B*.*pseudomallei* isolates of both clinical and environmental origin from distant geographical locations in the southern part of India.

Two major population groups depicting the STs of *B*.*pseudomallei* isolates from Southeast Asia and Australasia are observed currently on the eBURST population snapshot of MLST data (www.http://bpseudomallei.mlst.net/). Further, there are small clusters of STs reported from the rest of world either connected to Australasia and/or Southeast Asia or in the peripheries suggesting the possible transmission of the disease due to recent travel to the endemic areas. From the present study, we found that there is a considerable diversity among *B*. *pseudomallei* collected over a small spatial and temporal range in southern India, with 12 novel STs and 2 STs (42 and 960) that were previously reported on the MLST database. While, ST 1368 was the most prevalent genotype observed among our isolates, the ST 1372 was found to be the “group founder” for our study isolates. Unlike the Sri Lankan STs which formed a cluster between the Australasian and Southeast Asian clades, our study isolates were located in the periphery (outliers) of the eBURST population snapshot[11]. Relatedness of few Sri Lankan isolates as observed in the present eBURST population snapshot with the ST 1372 (group founder of our study isolates) suggest the possible dissemination of melioidosis across the two south Asian countries.

Despite the small number of isolates included in the present study, we observed: a) Indian isolates were distinct from the isolates reported from the rest of world (F_ST_ = 0.14413).b) Occurrence of the ST 1368 in three adjacent states of southern India (Karnataka, Kerala and Goa) suggesting the dispersion of *B*.*pseudomallei* across the south-western coastal part of India with limited geographical clustering. c) Lack of significant association between the STs and the form of the disease presentation. d) Occurrence of the infecting genotype (ST 1368) over a period of time and at different geographical locations suggesting the high levels of genetic uniformity at the housekeeping loci (low levels of nucleotide diversity,π = 0.0019) among the south-Indian *B*.*pseudomallei* isolates.
